# Impact of applying sex sorted semen on the selection proportion of the sire of dams selection pathway in a nucleus program

**DOI:** 10.5713/ajas.17.0108

**Published:** 2017-11-03

**Authors:** Sahereh Joezy-Shekalgorabi, Albert De Vries

**Affiliations:** 1 Department of Animal Science, Shahr-e-Qods Branch, Islamic Azad University, Tehran, Po Box: 37515-374, Iran; 2 Department of Animal Sciences, University of Florida, Gainesville, FL 32611, USA

**Keywords:** Sexed Semen, Selection Proportion, Sire of Dam Pathway, Heifer

## Abstract

**Objective:**

In a nucleus breeding scheme, the sire of dam’s pathway plays an important role in producing genetic improvement. Selection proportion is the key parameter for predicting selection intensity, through truncating the normal distribution. Semen sexing using flow cytometry reduces the number of vials of sperm that can be obtained from a proved bull. In addition, a lower fertility of this kind of sperm is expected because of the lower sperm dosage in sex sorted semen. Both of these factors could affect the selection proportion in the sire of dam’s pathway (*p* *_SD_*).

**Methods:**

In the current study, through a deterministic simulation, effect of utilizing sex sorted semen on selection (*p* *_SD_*) was investigated in three different strategies including 1: continuous use of sex sorted semen in heifers (CS), 2: the use of sex sorted semen for the first two (S2) and 3: the first (S1) inseminations followed by conventional semen.

**Results:**

Results indicated that the use of sex sorted semen has a negative impact on the sire of dams (SD) pathway due to increase in selection proportion. Consequently selection intensity was decreased by 10.24 to 20.57, 6.38 to 8.87 and 3.76 to 6.25 percent in the CS, S2, and S1 strategies, respectively.

**Conclusion:**

Considering the low effect of sexed semen on genetic improvement in dam pathways, it is necessary to consider the joint effect of using sex sorted semen on the sire and dams pathway to estimate about the real effect of sexed semen on genetic improvement in a nucleus breeding scheme.

## INTRODUCTION

Modifying the ratio of the X- bearing chromosome to the Y-bearing chromosome of bull semen by the flow cytometry technique has increased the chance for calving the desirable gender (usually female) in the last decade. The result of using this technology has been investigated for different genetic, reproductive and economic aspects [1–6]. The consequence of using sex sorted semen on genetic improvement of dairy cattle has been considered in several reports and simulation studies [5–12]. According to Van Vleck [13], a maximum of 15% increase in genetic improvement is expected in a dairy population when sex sorted semen is widely applied. In a dairy cattle breeding scheme using genomic selection, Pedersen et al [10] reported a maximum genetic gain of 6% in total merit index when sex sorted semen was applied in both nucleus and production populations. Pedersen et al [11] emphasized that the use of sex sorted semen has limited value on the genetic improvement compared with MOET. Khalajzadeh et al [14] reported an average superiority of 9.2% to 11.5% when applying sex sorted semen. They, however, did not find any considerable change in genetic superiority of active sires. Boustan et al [12], through a simulation study, predicted an increase of 25 and 34 to 38 percent, after applying sex sorted semen for traditional and genomic evaluation, respectively. Ghavi Hossein-Zadeh et al [9] studied genetic and economic impacts of using sex sorted semen by a stochastic model and demonstrated that the genetic improvement was greater in herds expanding the number of cows compared to herds with constant size. Abdel-Azim and Schnell [5] explained that using female-sorted semen in commercial herds, the average superiority of heifers exceeded 30%. They hypothesized that the increased merit is related to an increase in available heifers and hence enhanced selection intensity in the dam of dam’s pathway. The use of sex sorted semen in nucleus herds increased the genetic improvement in those herds. Joezy-shekalgorabi and Shadparvar [15], through a deterministic simulation, found that the use of sex sorted semen increased the genetic improvement of dam of dam pathway by about 5.82 to 39.65 percent.

Prediction of genetic improvement is an important stage in evaluating the effectiveness of a selection program. Rendel and Robertson [16] developed the formula for predicting genetic improvement in four different selection pathways (namely sire of sires [SS], sire of dams [SD], dam of sires [DS], and dam of dams [DD]), more than 60 years ago. Their proposed method could be applied for predicting the optimum genetic improvement in dairy breeding schemes. Sire of dam’s pathway considers the flow of genes from sire to their daughters (as future dams) in a commercial population. This pathway has a great impact on total genetic improvement in nucleus breeding programs [17]. This means that any change in genetic improvement on this pathway, will significantly affect the total genetic improvement. Selection intensity is one of the key parameters in predicting expected genetic improvement. Prediction of selection intensity is vital when defining the best selection and breeding programs. By applying the truncation selection method, it is possible to predict the selection intensity in various selection programs [18]. Considering the reverse relation of selection intensity and selection proportion, correct prediction of the selection proportion is necessary in a nucleus breeding program where various selection pathways are considered. Joezy-Shekalgorabi et al [17] presented the formulae for predicting the selection proportion in various selection pathways in a nucleus breeding program under a conventional progeny testing scheme. One of the advantages of using sex sorted semen is progeny testing of young bulls with a decreased test capacity [8].

The objective of the current study was to predict the selection proportion in SD pathway after applying their sex sorted semen for heifers. Considering that all the studies predicting genetic improvement after applying sex sorted semen have focused on dam pathway, this study could be important in designing the breeding strategies in nucleus herds, where sire pathways play an important role in improving the genetic makeup of the whole population. According to Joezy-Shekalgorabi et al [17], selection proportion in SD has direct relation with insemination number and reverse relation with the annual number of sperm dosage a proved bull produce. When using sex sorted semen from a bull, it is expected that he will have more daughters per insemination, but at the same time have fewer inseminations available and therefore fewer heifers can get pregnant. Considering this issue, semen sexing could affect the selection proportion in SD pathway. To follow this idea, we tried to simulate the effect of using sex sorted semen on the selection proportion in the sire of dam’s pathway by including the effect of sex sorted semen on both insemination number and the number of dosage obtained from a bull after semen sexing process in pure and mixed sexed semen strategies.

## MATERIALS AND METHODS

A deterministic simulation approach was utilized to study the effect of sex sorted semen in the SD pathway. We assumed that sex sorted semen was applied only for insemination of heifers. We assumed a constant population size during the selection program. In Iran only female sorted semen is available for insemination of heifers. Hence, in this study, we only considered the effect of sex sorted semen on the SD pathway. The effect of using sex sorted semen on selection proportion in this pathway was evaluated applying 3 breeding strategies including: i) continuous use of sexed semen (CS); ii) use of sexed semen for the first and second insemination and the use of conventional semen for the remaining inseminations (S2); iii) use of sexed semen for the first insemination followed by conventional semen for the second to the last insemination (S1). The accuracy of semen sorting was set to 90% female and 10% male [19] and the sex ratio for conventional semen was set to 50.8 and 49.2% for female and male calves, respectively [20]. Estrus detection rate (EDR) was set at 80%. The product of conception rate and estrus detection rate was defined as pregnancy rate. Number of services per conception, for reaching to a minimum cumulative pregnancy rate of 90% was estimated using the method described by Joezy-Shekalgorabi and Shadparvar [21].

Selection proportion in the sire of dam pathway was estimated according to Joezy-Shekalgorabi et al [17] with some changes to consider the effect of sex sorted semen in the model. The following equation was used for computing the selection proportion in the SD pathway (*p* *_SD_*).

$$p_{SD}=\frac{n\times {In}_{sex}\times (1-P)\times {rr}_{sd}}{t\times P\times {rr}_{f}\times d_{sex}}$$

Here, *n* refers to the number of daughters per young bull, *P* is the test capacity for the progeny testing program, t is the rate of cow population registered for milk recording in Iran and *rr* *_sd_* and *rr* *_f_* are replacement rates in SD pathway and in cows, respectively. The numeric values of these parameters for the typical situation in Iran are summarized in Table 1. The value of *d* *_sex_* and *In* *_sex_*, and consequently the *p* *_SD_*, was calculated depending on the applied strategy as follows:

For strategy CS:

$$\begin{array}{l} d_{sex}=FH\times \frac{d}{3.5}+(1-FH)\times d\\ {In}_{sex}=FH\times Inhsex+(1-FH)\times Inc \end{array}$$

For strategy S2:

$$\begin{array}{l} d_{sex}=FH\times \frac{d}{3.5}\times CPRates2+FH\times d\times (1-CPRates2)+(1-FH)\times d\\ {In}_{sex}=FH\times [Inhsex\times CPRates2+Inh\times (1-CPRates2)]+(1-FH)\times Inc \end{array}$$

For strategy S1:

$$\begin{array}{l} d_{sex}=FH\times \frac{d}{3.5}\times CPRates1+FH\times d\times (1-CPRates1)+(1-FH)\times d\\ {In}_{sex}=FH\times [Inhsex\times CPRates1+Inh\times (1-CPRates1)]+(1-FH)\times Inc \end{array}$$

Where, *d* = number of vials of sperm accessible from a proven bull per year; *FH* = the rate of heifers in the population; *Inc* = average number of service per conception in cows; *CPRates2* = cumulative pregnancy rate up to the second service in strategy S2; *CPRates* = cumulative pregnancy rate in the first service in strategy S1; *Inh* = number of services per conception in heifers after insemination with conventional semen; *Inhsex* = number of services per conception in heifers after insemination with sex sorted semen. The cumulative pregnancy rate was calculated according to formulas in Joezy-Shekalgorabi and Shadparvar [21].

The reason for dividing the number of doses of sperm (*d*) by 3.5 was that we assumed that for supplying a vial of sex sorted semen, about 3.5 vials of conventional semen was necessary (De Jarnette, personal communication).

Heifer conception rate using conventional semen and the proportion of the conception rate of sexed relative to conventional semen (*CRstoc*) varied from 50% to 90%. The conception rate of sex sorted semen was therefore obtained by multiplying the conventional semen conception rate and *CRstoc*. Also, in all scenarios, conception rate of conventional and sexed semen was assumed constant over consequent services. The *p* *_SD_* was estimated for the 3 strategies and for different values of conception rates. The results were compared to a control strategy (CC strategy), where no sex sorted semen was applied for heifers.

In order to simply estimate the *p* *_SD_* given the input parameters (i.e. *CRconh* and *CRstoc*), we also tried to find the optimum equation that fit equitably to the trend line obtained in various strategies.

## RESULTS AND DISCUSSION

**Figure 1:**
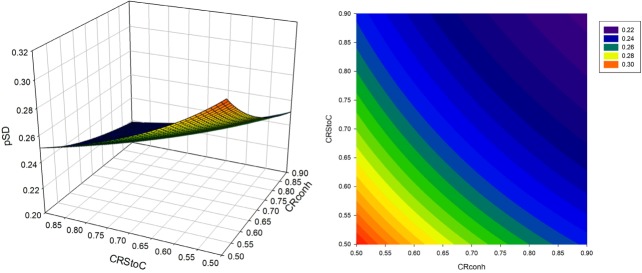
Selection proportion (*p*_SD_) in various conventional semen conception rates of heifers (*CRconh*) and the proportion of conception rate of sexed versus conventional semen (*CRstoc*) in CS strategy (continous utilization of sex sorted semen, in heifers).

**Figure 2:**
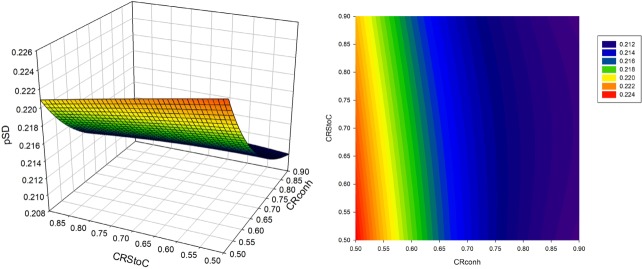
Selection proportion (*p*_SD_) in various conventional semen conception rates of heifers (*CRconh*) and the proportion of conception rate of sexed versus conventional semen (*CRstoc*) in S2 strategy (the use of sexed semen in the first and second insemination followed by conventional semen, in heifers).

**Figure 3:**
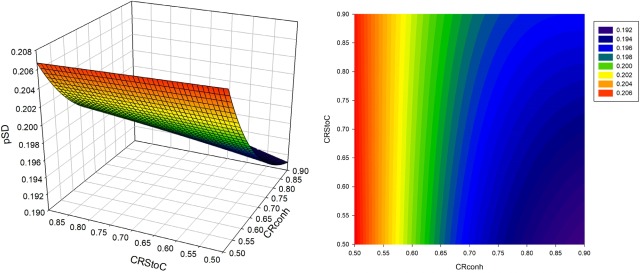
Selection proportion (*p*_SD_) in various conventional semen conception rates of heifers (*Crconh*) and the proportion of conception rate of sexed versus conventional semen (*CRstoc*) in S1 strategy (the use of sexed semen in the first insemination followed by conventional semen, in heifers).

Changes in *p* *_SD_* in sexed semen based strategies are illustrated via 3D plots and counter plots in Figures 1 to 3. The value of the *p* *_SD_* in all scenarios of the CS strategy was greater than that of the S2 and S1 strategies. The range of variation in *p* *_SD_* was about 21.84% to 30.9%, 21.02% to 22.43%, and 19.1% to 20.72% for strategies CS, S2 and S1, respectively. The value of *dsex* was constant when continuously utilizing of sex sorted semen. The values of *Insex* decreased when *CRstoc* and *CRconh* were increased. Due to the direct relation of between selection proportion and *Insex*, the trend of changes in *p* *_SD_* was similar to the trend for the number of services per conception. In the S2 strategy, the increase in conception rate of sexed versus conventional semen led to an initial increase followed by a subsequent decrease in the *p* *_SD_* (Figure 2) while in the S1 strategy, the increase in *CRstoc* led to an increase in the selection proportion. Different (direct and reverse) relations of *Insex* and *dsex* with *p* *_SD_* was the reason for different trend lines of S2 and S1 strategies, compared with CS strategy.

Equations fitted for predicting the *p* *_SD_* considering all effective parameters (*i.e.* conception rate of conventional semen in heifers, the rate of conception rate of sexed vs conventional semen) are presented in Table 2. All the strategies were equitably fitted to a second order paraboloid equation (adjusted *R* ^2^ was greater than 90%) which indicates the possibility of predicting *p* *_SD_* with a reasonable accuracy. The predicted trend line equations for the S2 and S1 strategies were more similar to each other, compared to the predicted trend line equation for the CS strategy.

**Figure 4:**
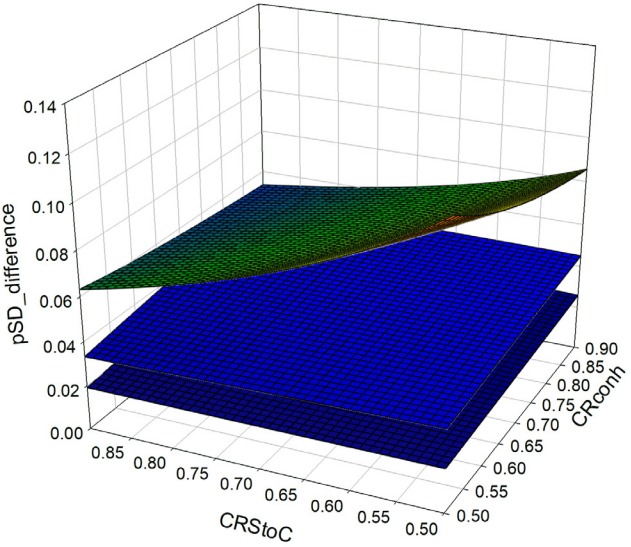
Difference of selection proportion in pure and mixed sexed semen strategies versus control strategy (continuous use of conventional semen).

Comparing the value of the selection proportion in the SD pathway in sexed semen based strategies with the CC strategy (conventional semen) indicated that more utilization of sexed semen enhances the selection proportion (Figure 4). The value of this increase was greater for the pure sexed semen strategy (CS) compared with the mixed sexed semen strategies (S2 and S1). The increase in the selection proportion in the sexed semen based strategies compared with control strategy was about 32.27% to 64.74% for CS strategy, 17.73% to 27.58% in S2 strategy and 10.19% to 18.92% in S1 strategy.

The increase in the selection proportion after applying sex sorted semen could be interpreted as to lower selection intensity and lower genetic improvement in the SD pathway. Considering the nature of the relationship between selection proportion and selection intensity in the truncation selection procedure, the extent of decrease in the selection intensity would not be the same with the extent of increase in selection proportion. As a result, the value of a decrease in selection intensity in strategies CS, S2, and S1 was about 10.24% to 20.57%, 6.38% to 8.87%, and 3.76% to 6.25%, respectively.

The reason for declining selection intensity in the SD pathway is the decline of number of vials of sperms that could be obtained from each proven sire. In addition, due to a lower conception rate expected from sex sorted semen, a greater number of sires are necessary for inseminations of heifers. The increase in the number of sires needed in the SD pathway increases the selection proportion and subsequently, decreases selection intensity and expected genetic improvement. In most studies about the effect of sex sorted semen, the emphasis is on the importance of using sex sorted semen on dam of dams pathways and its effect on increasing genetic improvement in that pathway [9,14].

Contrary to the result of previous studies [5,9,12], it is expected that the consequence of using sex sorted semen on genetic improvement would be a decline in genetic improvement in a nucleus breeding scheme.

It should be noted that in the current study, we assumed that all of available doses of sperm in the SD pathway is consumed for producing sex sorted semen. While in practice, both sex sorted and conventional semen is available for a bull that is considered as sire of dams. Hence it is expected that in practice, considering the rate of using sex sorted versus conventional sperm, the value of a decrease in selection intensity would be somewhat lower compared with the results of our study such that this decrease may be covered by the increase in genetic gain in dam pathways. To estimate the advantages of using sex sorted semen, it is necessary to include all aspects of using this technology.

## IMPLICATIONS

The results of the current study indicated that the use of sex sorted semen has a negative impact on selection proportion and genetic improvement in the sire of dam’s pathway. However, we did not take into account the possibility of using sex sorted semen in young bulls (as potential sire of dams) in the progeny testing program. Besides, to conclude about the effect of sex sorted semen on total genetic improvement we need to consider the joint effect of using this kind of sperm in both sires and dams pathways, concurrently. On the other hand, to decide about the possible benefit of sex sorted semen it is necessary to consider the economic efficiency of using this kind of semen as well as its effect on genetic improvement.

## Figures and Tables

**Table 1:** Utilized parameters for simulation of nucleus breeding program

Parameter	Abbreviation	Value
Percent of cows on milk recording	*t*	33
Percent of cows inseminated with young bull semen	*P*	20
Progeny group size	*n*	100
Number of insemination per pregnancy in dairy cows	*Inc*	2.58
Number of doses of semen used by each progeny tested bulls	*d*	18,000
Replacement rate of sire of daughters	*rr*_sd_	0.25
Cow replacement rate	*rr*_f_	0.2118
Percent of heifers in the population	*FH*	32

**Table 2:** Adjusted equations for predicting selection proportion in SD pathway (*p*_SD_) considering various conception rate of conventional semen (x) and the rate of conception rate of sexed versus conventional semen (y)

Strategy1)	Equation fitted	Adjusted R^2^	SE
CS	*p*_SD_= 0.5385−0.3160x−0.3160y+0.1516x^2^+0.1516y^2^	0.9843	0.0022
S2	*p*_SD_= 0.2667−0.124x+0.0049y+0.0686x^2^−0.0059y^2^	0.99	0.0004
S1	*p*_SD_= 0.2558−0.1472x−0.0045y+0.0827x^2^+0.0009y^2^	0.9817	0.0005

SE, standard error.

1) CS, continuous use of sexed semen; S2, use of sexed semen for the first and second insemination and the use of conventional semen for the remaining inseminations; S1, use of sexed semen for the first insemination followed by conventional semen for the second to the last insemination.
